# Diagnosing Noise-Induced Hearing Loss Sustained During Military Service Using Deep Neural Networks

**DOI:** 10.1177/23312165231184982

**Published:** 2023-08-07

**Authors:** Brian C.J. Moore, Josef Schlittenlacher

**Affiliations:** 1 Cambridge Hearing Group, Department of Psychology, University of Cambridge, Cambridge, UK; 2 Department of Speech, Hearing and Phonetic Sciences, 4919 University College London, London, UK

**Keywords:** hearing loss, noise exposure, neural network, diagnosis

## Abstract

The diagnosis of noise-induced hearing loss (NIHL) is based on three requirements: a history of exposure to noise with the potential to cause hearing loss; the absence of known causes of hearing loss other than noise exposure; and the presence of certain features in the audiogram. All current methods for diagnosing NIHL have involved examination of the typical features of the audiograms of noise-exposed individuals and the formulation of quantitative rules for the identification of those features. This article describes an alternative approach based on the use of multilayer perceptrons (MLPs). The approach was applied to databases containing the ages and audiograms of individuals claiming compensation for NIHL sustained during military service (M-NIHL), who were assumed mostly to have M-NIHL, and control databases with no known exposure to intense sounds. The MLPs were trained so as to classify individuals as belonging to the exposed or control group based on their audiograms and ages, thereby automatically identifying the features of the audiogram that provide optimal classification. Two databases (noise exposed and nonexposed) were used for training and validation of the MLPs and two independent databases were used for evaluation and further analyses. The best-performing MLP was one trained to identify whether or not an individual had M-NIHL based on age and the audiogram for both ears. This achieved a sensitivity of 0.986 and a specificity of 0.902, giving an overall accuracy markedly higher than for previous methods.

## Introduction

It is well known that exposure to intense sounds for a sufficient time can lead to noise-induced hearing loss (NIHL) and this is recognized in safety standards and workplace regulations (National Institute for Occupational Safety and Health, 2020; European Parliament, 2003). Accurate diagnosis of NIHL is important for individuals who wish to claim compensation for NIHL sustained during their employment. Almost all methods of diagnosing NIHL are based largely on the audiogram, the hearing threshold level (HTL) measured as a function of frequency. Where reliable audiograms are available before and after a period of noise exposure, then changes in HTL over time that are greater than the changes expected from age alone can be used as evidence that NIHL has occurred. For example, according to the guidelines of the US Department of Defence (2019), NIHL is deemed to be present when there is “a change in hearing threshold relative to the baseline audiogram of an average of 10 dB or more at 2000, 3000, and 4000 Hz in either ear.” However, the reliability and validity of occupational audiograms is questionable (Lowe & Moore, 2021).

An alternative approach is to base the diagnosis of NIHL on one or more audiograms obtained after the period of noise exposure has occurred. Many such methods involve identification of a notch or bulge in the audiogram centered near 4 kHz (Coles et al., 2000; Niskar et al., 2001; Phillips et al., 2010; Pudrith et al., 2022). Notches typically occur following exposure to intense steady broadband noise, such as occurs in some factories (Passchier-Vermeer, 1974; Smoorenburg, 1992). However, Moore (2020) argued that methods involving notch identification are not appropriate in cases of NIHL sustained during military service, hereafter denoted M-NIHL. Military service often involves exposure to very intense impulsive sounds, which are more damaging than steady sounds with the same energy (Zhang et al., 2021; Zhang et al., 2022). M-NIHL is sometimes associated with a notch or bulge in the audiogram, but sometimes is associated with a hearing loss that increases progressively with increasing frequency, without any clear notch (Moore, 2020; Lowe & Moore, 2021). Also, M-NIHL can occur over a wide range of frequencies and, when a notch is present, it may not be centered at 4 kHz (Keim, 1969; Moore, 2020; Lowe & Moore, 2021).

Moore (2020) proposed a diagnostic method, called here the M-NIHL (2020) method, that essentially made a positive diagnosis of M-NIHL based on the satisfaction of either or both of two requirements (in addition to the requirement of evidence for noise exposure sufficient to have the potential for causing NIHL): (1) there should be a notch or bulge in the audiogram of a certain magnitude centered at 3, 4, or 6 kHz; (2) there should be a hearing loss at high frequencies (4, 6, or 8 kHz) exceeding the loss that would be expected from age alone. That method was shown to have high sensitivity (the proportion of individuals with M-NIHL who receive a positive diagnosis) but only moderate specificity (the proportion of non-noise-exposed individuals who are correctly diagnosed as not having M-NIHL) (Moore & von Gablenz, 2021). Moore et al. (2022b) proposed a revision of the M-NIHL (2020) method, called the rM-NIHL method, aimed at achieving markedly improved specificity at the expense of a small reduction in sensitivity. The modifications included increasing the magnitude of the notch depth required for notch identification and assessing high-frequency hearing loss by averaging across the frequencies 4, 6, and 8 kHz. For the case where an individual is classified as having NIHL based on a positive diagnosis for either or both ears, the rM-NIHL method was estimated to have a sensitivity of 0.979 and a specificity of 0.630, giving a detectability index, *d′*, of 2.37.

All audiogram-based approaches to the diagnosis of NIHL developed so far have involved examination of the typical features of the audiograms of noise-exposed individuals and then the formulation of quantitative rules for the identification of those features. This article describes an alternative approach based on the use of deep neural networks, specifically multilayer perceptrons (MLPs). Rather than relying on researchers identifying the features of the audiogram that characterize M-NIHL, the MLPs were trained so as to classify individuals as having or not having M-NIHL based on their audiograms and ages, thereby automatically identifying the features of the audiogram that provide optimal classification.

The approach using MLPs is based on the availability of databases of the audiograms of noise-exposed individuals who are likely to have NIHL and control databases of individuals with no known exposure to intense sounds, but who are matched in other respects. In this article, the databases described by Moore et al. (2022b) were used; these are described in more detail later in this article. There were four databases in total, two of which were used for training and validation of the MLPs and two of which were used for testing. One of the training databases, denoted MilDB1, was composed of the audiograms and ages of former military personnel who were claiming compensation for M-NIHL. It was assumed that most of these individuals did have M-NIHL. The second training database, denoted ContDB1, was composed of the audiograms and ages of individuals without known exposure to intense noise, who were matched in other characteristics to the individuals in MilDB1. It was assumed that these individuals did not have any form of NIHL. The MLPs were trained to use the audiograms and ages in the databases to categorize each individual as belonging to the noise-exposed group or the nonexposed group. Once the MLPs were trained, their classification accuracy was assessed using the remaining two databases, MilDB2 and ContDB2, which were very similar to MilDB1 and ContDB1, but were based on different individuals.

In previous work on the diagnosis of NIHL, the diagnostic methods have been applied separately to each ear. An individual has been deemed to have NIHL if the requirements of a given diagnostic method were met for either ear or both ears. In the present article, a different approach is evaluated where the MLP is supplied with information about age and the audiogram for both ears, and uses all of that information to classify an individual as having or not having M-NIHL.

An MLP can be trained so as to prioritize sensitivity over specificity or vice versa. If a diagnosis is made in the context of a claim for compensation for NIHL, then it is important to achieve high sensitivity, so as to avoid unfairly denying compensation to an individual who does have M-NIHL. However, specificity should be sufficiently high to avoid compensation being given to a high proportion of individuals who do not have NIHL. In a medicolegal context, for a positive diagnosis to be made it should be more likely than not that the individual has NIHL. This is often referred to as the “balance of probabilities.” However, this balance depends on several factors other than age and the audiogram, including the “true” prevalence of NIHL among the noise-exposed population that is being considered, and this is unknown. Later in this article, we give estimates of positive predictive value (PPV) for the best-performing MLP and also for previous diagnostic methods. The PPV indicates how likely it is for someone in the population of claimants to truly have M-NIHL in the case of a positive diagnosis using a given method.

To make a diagnosis of M-NIHL, it is necessary to assess whether there is any plausible cause of hearing loss other than noise exposure during military service, including identifiable ear diseases and noise exposure outside military service (Moore et al., 2022a). For all of the individuals contributing to MilDB1 and MilDB2, a thorough medical history had been obtained and causes of hearing loss other than noise exposure during military service had been deemed to be unlikely.

## Method

### Study Populations

The databases used in this work were the same as described in detail by Moore et al. (2022b), and only summary information is given here. Databases MilDB1 (143 individuals) and MilDB2 (142 individuals), used for training/validation and testing of the MLPs, respectively, contained the ages and audiograms of men who were claiming compensation for M-NIHL sustained during service in the British military. Each of these individuals had approached a legal company to handle their claim, and the legal companies in turn had requested a medical report on each claimant, including an audiogram. Inclusion in MilDB1 and MilDB2 did not depend on the outcome of the claim or on whether the author of the medical report did or did not diagnose M-NIHL. The ages of the individuals ranged from 29 to 60 years. All reported exposure to intense impulsive sounds, sometimes without hearing protection. Over 80% reported times when they had a temporary dulling of hearing and/or tinnitus following such exposure, which is often taken as an indicator of exposure to noise with the potential to cause hearing loss. Audiograms obtained before or close to the start of military service indicated no ear asymmetry ≥10 dB (based on the average HTL across 0.5, 1, 2, 3, 4, and 6 kHz). Estimates of sensitivity were based on the assumption that the great majority of ears in MilDB1 and MilDB2 had M-NIHL, which may not have been the case. Hence, the true sensitivity is likely to be higher than estimated. However, errors in the estimate of sensitivity in either direction could occur if the proportion of individuals who had N-MIHL differed for MildB1 and MildB2.

Databases ContlDB1 (93 individuals) and ContDB2 (92 individuals), used for training/validation and testing of the MLPs, respectively, were based on a control population (von Gablenz & Holube, 2016) screened to exclude significant noise exposure (Moore & von Gablenz, 2021). The age range was the same as for the MilDB1 and MilDB2 databases. It was assumed that the great majority of individuals in ContDB1 and ContDB2 did not have NIHL, although these individuals were not medically examined, so some of them may have had causes of hearing loss other than noise exposure.

Means and standard deviations (SDs) of the HTLs for each database are given in Table 1 of Moore et al. (2022b). On average the individuals in MilDB1 and MilDB2 had greater hearing loss at high frequencies for the left ears than for the right ears, as has been reported previously for M-NIHL (Keim, 1969; Moore, 2020; Lowe & Moore, 2021). This is probably mainly due to greater noise exposure of the left ear, produced, for example, by the way that a rifle is usually fired from the right shoulder, which results in partial shielding of the right ear via the head-shadow effect (Keim, 1969). Engdahl and Aarhus (2023) assessed ear asymmetry for two large cohorts of people who used recreational firearms and found that ear asymmetry was smaller for the recent cohort, consistent with the idea that the asymmetry is related to greater exposure of the left ear, on average, rather than to inherent differences of the left and right ears in susceptibility to NIHL. Because of this ear asymmetry, it was anticipated that for MLPs based on use of the audiogram from one ear only, accuracy would be greater for the left than for the right ears.

**Table 1.** Validation Accuracies, Averaged Across the 10 Runs of the Cross Validation and All MLPs With Different Numbers of Hidden Layers and Hidden Units.

Number of features	Input features	Validation accuracy
6	HTL right	0.868
6	HTL left	0.908
6	ACHTL right	0.888
6	ACHTL left	0.912
12	HTL right and AAHL	0.883
12	HTL left and AAHL	0.911
12	HTL right and HTL left	0.921
12	ACHTL right and ACHTL left	0.923
18	AAHL, HTL right and HTL left	0.925

AAHL: age-associated hearing loss; ACHTL: age-corrected HTL; HTL: hearing threshold level; MLP: multilayer perceptron.

### Features Used as Input to the MLPs

Several MLPs were trained, each using a specific set of input features. The values of all input features were z-score normalized by subtracting the mean across all instances of the training set, MilDB1 and ContDB1, and dividing by the SD for each feature. The means and SDs of the training set were also used for evaluations based on the test databases (MilDB2 and ContDB2). Differences between the measured HTLs and the corresponding age-associated hearing loss (AAHL) values are denoted age-corrected HTLs (ACHTLs). AAHL values were taken from ISO 7029 (2017). These AAHL values are also used within the M-NIHL (2020) and rM-NIHL methods.

To allow comparison with previous diagnostic methods for M-NIHL, some of the MLPs used features for one ear only. These features were:

1. HTLs for the right ear at 1, 2, 3, 4, 6, and 8 kHz (6 features).
2. HTLs for the left ear at 1, 2, 3, 4, 6, and 8 kHz (6 features).
3. ACHTLs for the right ear at 1, 2, 3, 4, 6, and 8 kHz (6 features).
4. ACHTLs for the left ear at 1, 2, 3, 4, 6, and 8 kHz (6 features).
5. HTLs for the right ear and AAHLs (12 features).
6. HTLs for the left ear and AAHLs (12 features).

The last two were intended to capture possible interaction and combination effects of HTLs and AAHLs.

It was likely that the provision of information about the two ears would allow an MLP to learn from the data so as to give better diagnostic accuracy for an individual than when using features for each ear separately. To assess whether this was the case, three further MLPs with twelve or eighteen input features were trained. These were:

1. HTLs for the left and right ears (12 features).
2. ACHTLs for the left and right ears (12 features).
3. AAHLs and HTLs for the left and right ears (18 features).

### MLP Architecture

MLPs with between one and five hidden layers and two and five hidden units in each layer were evaluated in an exhaustive search. The activation function for the hidden units was the hyperbolic tangent and the activation function for the single output unit was the sigmoid.

### Training and Validation of the MLPs

The MLPs were trained to minimize the binary cross-entropy loss of the output unit. This optimizes accuracy, which is defined as the overall proportion of cases classified correctly. To give slightly greater weight to sensitivity than to specificity, which, as discussed in the Introduction section, is appropriate in a medicolegal context, a class weighting of three was given to MilDB1 relative to ContDB1. In other words, MilDB1 was used three times as often as ContDB1 during training of the MLP. Note that this weighting to prioritize sensitivity over specificity was in addition to the imbalance in numbers of individuals in the two databases (143 for MilDB1 and 93 for ContDB1).

MLPs for each setting of the hyperparameters (choice of input features, hidden layers, and hidden units) were trained in a 10-fold cross validation, where in each run 14 randomly selected but different cases from MilDB1 and 9 from ContDB1 were used to calculate the validation accuracy and the rest (129 in MilDB1 and 82 in ContDB1) were used to train the MLP. Validation accuracies were averaged across the 10 training runs.

The MLPs were trained using the Adam optimizer (Kingma & Ba, 2014) with a learning rate of 0.0001. This optimizer is the default in many computing packages and uses an extended version of stochastic gradient descent; other optimizers are likely to produce similar results. The chance of overfitting was reduced by using L2 normalization (Krogh & Hertz, 1991) with a weight of 0.01 (i.e., favoring small weights by adding the sum of all squared weights, multiplied by 0.01, to the training error). Training stopped after 10 epochs without any improvement in the validation error or after 500 epochs, whichever occurred earlier.

### Analysis and Choice of Best MLP

For comparison with previous methods for diagnosing M-NIHL and to investigate if there are differences between the left and right ears, the validation outcomes for the single-ear MLPs were examined first. It was also assessed whether the use of ACHTL values led to more accurate predictions than the use of HTLs. The effects of these two factors were studied in an analysis of variance (ANOVA) that also included the factors “number of hidden units” and “number of hidden layers.” If neither “number of hidden units” nor “number of hidden layers” is significant, and if there is no interaction between the two, this justifies use of the simplest MLP architecture, with the minimum number of hidden units and layers. Otherwise, the configuration with the highest validation accuracy should be considered the best MLP configuration.

Among all MLPs, including those with more input features, the MLP with the highest validation accuracy was selected as the best choice and was analyzed in more detail.

## Results

### Validation Accuracies and Effects of Number of Hidden Layers and Units

The grand means of the validation accuracies for all nine sets of input features are shown in Table 1. Of the four MLPs with six input features (i.e., six measures for one ear), accuracy was greater for the left ear than for the right ear, by 2 to 4 percentage points, as expected given the greater average hearing losses for the left ears in MilDB1. Use of the ACHTL values gave slightly higher accuracy than use of the HTLs, by 0.4 percentage points for the left ear and 2 percentage points for the right ear. A four-way ANOVA (ear × threshold measure × number of hidden layers × number of hidden units) of the validation accuracies for these four MLPs showed significant main effects of ear, *F*(1,540) = 44.9, *p* < 0.001, and threshold measure (ACHTL vs. HTL), *F*(1,540) = 4.82, *p* < 0.05. The interaction between ear and threshold measure approached but did not reach significance, *F*(1,540) = 3.45, *p* = 0.06. All other *F* values were smaller than 0.5, suggesting that the smallest number of hidden layers and hidden units was sufficient.

To verify that a greater number of hidden layers and hidden units did not lead to improved accuracy, a three-way (input feature set × number of hidden layers × number of hidden units) ANOVA was also calculated for the five MLPs with 12 or 18 input features. The main effect of input feature set was significant, *F*(4,900) = 10.8, *p* < 0.001. All other *F* values were smaller than 0.2 with *p* values higher than 0.9.

The lack of improvement produced by greater numbers of hidden layers and hidden units is also apparent in the descriptive statistics. The MLP with the highest average validation accuracy, that with 18 input features, achieved its highest validation accuracy (0.939) for an MLP with five layers each with four units and its lowest validation accuracy (0.909) for an MLP with five layers with five units. The validation accuracy for an MLP with one hidden layer and two hidden units was only slightly different, at 0.926.

**Figure 1.**
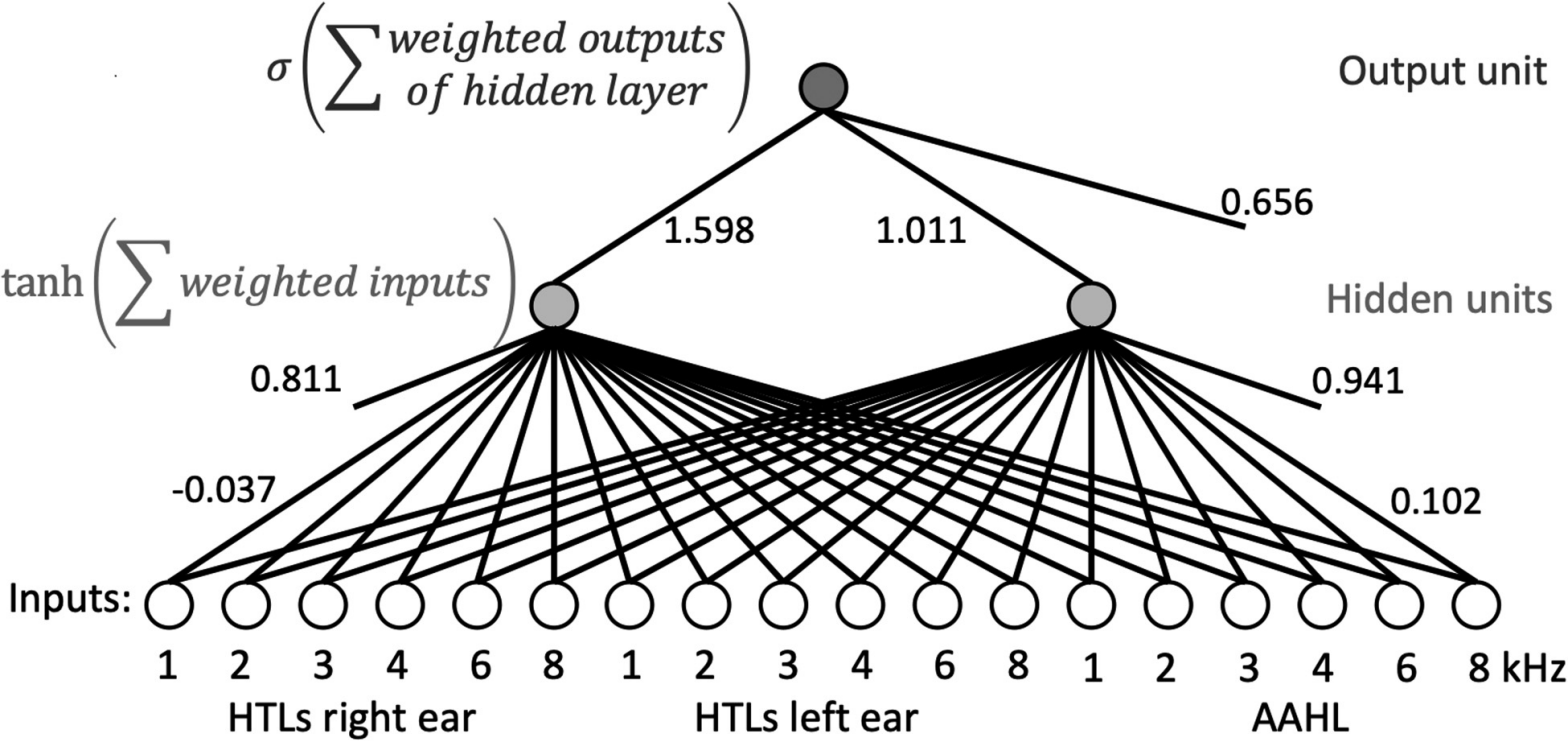
Schematic diagram of MLP(18). White circles (bottom) contain the input values from which a diagnosis is to be made. Each line is associated with a weight, by which its input from the preceding node is multiplied before being summed by the node that it connects to. For clarity, the values are shown only for a few weights; see Table 2 for all weights. Weights that are not connected to an input are bias weights. The mathematical operations that are performed by the two hidden units (center) and output unit (top) are indicated on the left. MLP: multilayer perceptron.

These outcomes led us to choose the MLP with 18 inputs, one hidden layer and two hidden units for further analysis. Note that this MLP is intended to diagnose whether an individual has M-NIHL and not whether each ear has M-NIHL. The weights for this trained MLP, hereafter denoted MLP(18) are given in Table 2. These include the bias weights, which are constants that are added to the sum of all other inputs. They are all positive numbers, which was to be expected when favoring sensitivity over specificity. Figure 1 shows a schematic diagram of the MLP, where each line represents a weight that is given in Table 2. The weights are interpreted in the Discussion section.

**Table 2.** Weights of MLP(18), Which had 18 Input Features.

	From input	To hidden unit 1	To hidden unit 2	From	To output
HTL right	1 kHz	−0.037	0.129	Unit 1	1.598
	2 kHz	0.113	0.213	Unit 2	1.011
	3 kHz	0.061	0.236	Bias	0.656
	4 kHz	0.470	0.376		
	6 kHz	0.421	0.136		
	8 kHz	−0.337	0.097		
HTL left	1 kHz	0.689	0.368		
	2 kHz	−0.009	−0.090		
	3 kHz	−0.127	0.079		
	4 kHz	0.436	0.621		
	6 kHz	0.507	0.136		
	8 kHz	−0.009	0.035		
AAHL	1 kHz	0.224	−0.138		
	2 kHz	−0.332	0.079		
	3 kHz	−0.239	−0.189		
	4 kHz	−0.065	−0.014		
	6 kHz	0.064	0.097		
	8 kHz	0.126	0.102		
	Bias	0.811	0.941		

AAHL: age-associated hearing loss; HTL: hearing threshold level; MLP: multilayer perceptron.

It should be noted that the weights in Table 2 are for one specific trained network. Slightly different weights (and slightly different accuracies) would be obtained if the network were trained again, because of the random initialization of the weights and the limited amount of training data. To assess the effect of this variability on the accuracy of the predictions, MLP(18) was trained 20 times, each with a different random initialization of the weights. The mean obtained sensitivity was 0.983 with a SD of 0.006 and the mean specificity was 0.905 with an SD of 0.022. Thus, the variability was small. The randomly selected MLP(18) whose weights are shown in Table 2 was saved and used for further analysis.

### Comparison With Previous Diagnostic Methods for M-NIHL

Table 3 shows sensitivity, specificity and *d’* values for the M-NIHL (2020) method (Moore, 2020), the rM-NIHL method (Moore et al., 2022b), and the MLP(18) method developed in this article, all based on diagnosis of the individual (either or both ears for the M-NIHL (2020) and rM-NIHL methods) and using the test databases MilDB2 and ContDB2. Note that MilDB2 and ContDB2 were not used in the training or validation of the MLP(18) method. The MLP(18) method led to very slightly lower sensitivity than for the M-NIHL (2020) method, but to slightly higher sensitivity (by 1 percentage point) than for the rM-NIHL method. The MLP(18) method led to markedly higher specificity than for the M-NIHL (2020) and rM-NIHL methods, and the discriminability index, *d’* was markedly higher for the MLP(18) method than for the two other methods. Overall, the MLP(18) method clearly had higher diagnostic accuracy than the other two methods.

**Table 3.** Sensitivity, Specificity, and *d’* Values for the M-NIHL (2020) Method (Moore, 2020), the rM-NIHL Method (Moore et al., 2022b), and the MLP(18) Method Developed in This Article, All Based on Diagnosis of the Individual (Either or Both Ears for the M-NIHL (2020) and rM-NIHL Methods).

Method	Sensitivity	Specificity	*d'*
M-NIHL (2020)	0.993	0.402	2.21
rM-NIHL	0.976	0.639	2.37
MLP(18)	0.986	0.902	3.49

MLP: multilayer perceptron; M-NIHL: military service noise-induced hearing loss; rM-NIHL: revision of M-NIHL (2020).

### Positive Predictive Values

The PPV indicates how likely it is for someone in the population of claimants for compensation for M-NIHL to truly have M-NIHL in the case of a positive diagnosis using a given method (Fletcher, 2019). The PPV is defined as

PPV = (sensitivity × prevalence)/[(sensitivity × prevalence) + ((1 – specificity) × (1 – prevalence))],

where prevalence here refers to the prevalence of M-NIHL among the population of claimants. The prevalence is, of course, unknown, but is likely to be reasonably high, and almost certainly above 0.5 (Moore, 2023). The assumption of a reasonably high prevalence is consistent with the high diagnostic accuracy of the MLP(18) method.

The value of the PPV ranges from 0 to 1. A value >0.5 indicates that the “balance of probabilities” is met. Table 4 shows sensitivity, specificity, and PPV values for the M-NIHL (2020) method, the rM-NIHL (2020) method, and the MLP(18) method, all based on diagnosis of the individual (either or both ears for the M-NIHL (2020) and rM-NIHL methods) and using the test databases MilDB2 and ContDB2. Three prevalence values were considered, one (0.9) assumed to be reasonably realistic, the second (0.5) intended to represent a plausible lower bound, and the third (0.25) implausibly low. For the assumed prevalence values of 0.9 and 0.5, all methods gave PPV values above 0.5, indicating that the balance of probabilities was met. The MLP(18) method gave higher PPV values than the two other methods, especially for the assumed prevalences of 0.5 and 0.25. For the prevalence of 0.5, the PPV was only a little above 0.5 for the M-NIHL (2020) method. For the implausibly low assumed prevalence of 0.25, the PPV was below 0.5 for the M-NIHL (2020) and rM-NIHL methods but remained well above 0.5 (at 0.770) for the MLP(18) method.

**Table 4.** PPV Values for the M-NIHL (2020) Method, the rM-NIHL Method, and the MLP(18) Method, All Based on Diagnosis of the Individual, Using Assumed Prevalence Values of 0.9, 0.5, and 0.25.

Method	Assumed prevalence	PPV
M-NIHL (2020)	0.9	0.937
rM-NIHL	0.9	0.960
MLP(18)	0.9	0.989
M-NIHL (2020)	0.5	0.625
rM-NIIHL	0.5	0.683
MLP(18)	0.5	0.910
M-NIHL (2020)	0.25	0.356
rM-NIIHL	0.25	0.469
MLP(18)	0.25	0.770

MLP: multilayer perceptron; M-NIHL: military service noise-induced hearing loss; PPV: positive predictive value; rM-NIHL: revision of the M-NIHL.

It can be concluded that for plausible prevalence values all three diagnostic methods give PPV values above 0.5, thereby satisfying the balance of probabilities required in a medicolegal context, but that the MLP(18) method is more robust, giving a PPV above 0.5 even when the prevalence among the population of claimants is assumed to be unrealistically low. Also, the MLP(18) method has the benefit of having markedly higher specificity than the M-NIHL (2020) and rM-NIHL methods, meaning that use of the MLP(18) method will only rarely give a positive diagnosis of M-NIHL for an individual who does not have M-NIHL.

## Discussion

The MLP(18) method gave higher overall accuracy than the M-NIHL (2020) or rM-NIHL diagnostic methods when diagnosing an individual as having or not having M-NIHL. This could have occurred because the MLP(18) used patterns in the data that were informative but were not used by the M-NIHL (2020) or rM-NIHL diagnostic methods. Alternatively, or in addition, the superior diagnostic accuracy of the MLP(18) method might reflect the fact that it uses information about both ears, whereas the M-NIHL (2020) or rM-NIHL methods are applied separately to the left and right ears, and an individual is deemed to have M-NIHL if the diagnosis is positive for either or both ears. To assess whether the use of MLPs gives higher diagnostic accuracy than previous methods when applied to each ear separately, MLPs based on HTLs and AAHLs for the right ear only and on HTLs and AAHLs for the left ear only were used to evaluate MilDB2 and ContDB2, and an individual was deemed to have M-NIHL if the diagnosis was positive for either or both ears. The resulting sensitivity and specificity values were 1 and 0.761, respectively. These values are higher than for the M-NIHL (2020) method and the rM-NIHL method (see Table 3). Hence, it can be concluded that the superiority of the MLP(18) method was at least partly due to it being able to exploit patterns in the data that were not used by the M-NIHL (2020) or rM-NIHL diagnostic methods.

A problem with the application of the MLP(18) method is that it is difficult to interpret exactly how it works. The weights shown in Table 1 suggest that HTLs at 4 and 6 kHz in both ears are highly weighted, which makes sense since noise exposure often has its greatest effects at these frequencies (Humes et al., 2006; Moore, 2020; Lowe & Moore, 2021). For the left ear only, the HTL at 1 kHz had a high weight. This may reflect the fact that as NIHL becomes more severe it tends to spread to frequencies well away from 4 to 6 kHz (Passchier-Vermeer, 1974; Lowe & Moore, 2021). Some of the weights that lead to the first hidden unit, for example, at 8 kHz, are negative, which could indicate that this unit looks for a notch at 4 and 6 kHz rather than just a large hearing loss. The same might apply to the interaural comparison of the HTLs at 1 kHz, where the left ear has the highest weight of the whole network while the right ear has a slightly negative weight. Other than that, there seem to be no clear differences between weights for the two ears, possibly due to a higher threshold for both ears being somewhat more indicative of NIHL than a particular asymmetry. There are no obvious interactions originating from the AAHL, which may be partly because it encodes a single variable, age. The AAHL weights are mostly negative, probably reflecting the fact that a higher HTL is to be expected for older people, independent of their noise exposure.

**Figure 2.**
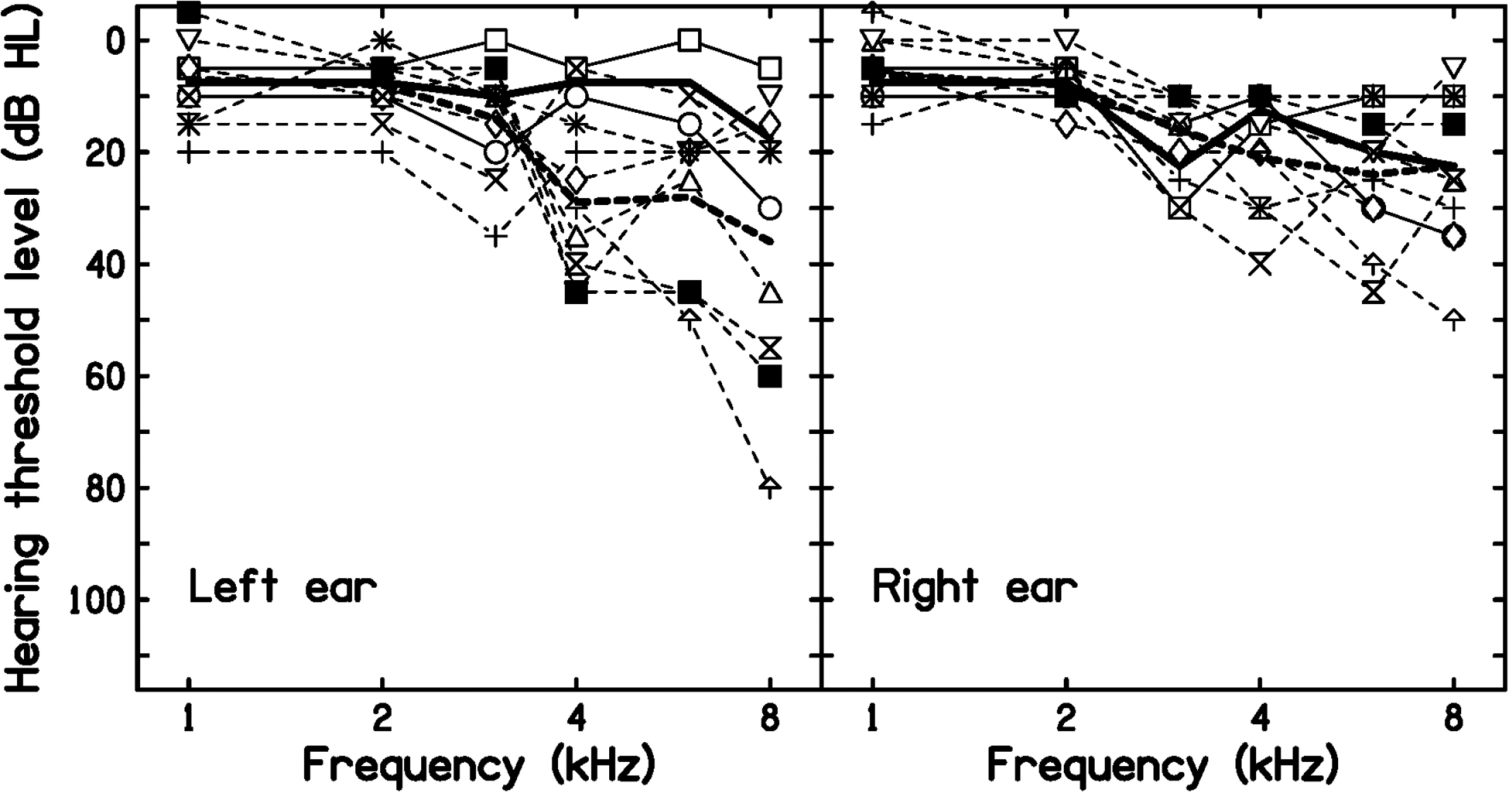
Audiograms for the left ears (left panel) and right ears (right panel) of individuals in MilDB2 who received a negative diagnosis using MLP(18) (solid lines, thick lines indicating the mean), and individuals in ContDB2 who received a positive diagnosis (dashed lines, thick lines indicating the mean). A different symbol is used for each individual. MLP: multilayer perceptron.

It is instructive to consider the few cases where MLP(18) made a negative diagnosis for individuals in MilDB2 and a positive diagnosis for individuals in ContDB2. The audiograms for these cases are shown by solid and dashed lines, respectively, in Figure 2. The two individuals in MilDB2 who received a negative diagnosis (open squares and open circles) both had only mild hearing loss; their primary complaint was tinnitus rather than hearing difficulties. The individuals in ContDB2 who received a positive diagnosis mostly had greater hearing loss at high frequencies for the left than for the right ear, as shown by the thick lines indicating the means in Figure 2. This suggests that MLP(18) was exploiting the fact that in cases of M-NIHL the hearing loss is often greater for the left than for the right ear (Keim, 1969; Moore, 2020; Lowe & Moore, 2021). This ear asymmetry is not taken into account by the M-NIHL (2020) or rM-NIHL methods. A few but not all of the individuals in ContDB2 who received a positive diagnosis had notches in their audiograms in one or both ears at 3, 4, or 6 kHz, suggesting that MLP(18) was also using audiometric notches as a diagnostic marker.

## Implementation

MLPs are usually implemented in specialized programming languages in which they are trained, such as Python, and many potential users of MLP(18) will not be familiar with such software. However, MLP(18) is sufficiently simple to allow an implementation in Excel, which is widely available and easy to use. Such an Excel implementation is available upon request from either of the authors.

## Limitations

Some limitations of this study are the same as those for the study of Moore et al. (2022b) on the rM-NIHL method, so they will be addressed only briefly here. Firstly, MilDB1 and MilDB2 were restricted to those claiming compensation for M-NIHL. This increased the likelihood of them having M-NIHL, making them suitable for estimating the sensitivity of the MLP methods, but with the risk that the hearing loss was exaggerated (Moore, 2023). However, all of the audiograms in MilDB1 and MilLDB2 were obtained according to the recommendations of the British Society of Audiology (2018), which meant that the procedure incorporated measures of response consistency. Another limitation is that the noise-exposed and control samples were not matched in terms of alcohol consumption, smoking, socio-economic status, or educational level, all of which are weakly associated with HTLs (Davis, 1995; Dawes et al., 2014). Finally, the individuals whose audiograms were in MilDB1 and MilDB2 were probably less highly screened than those in ContDB1 and ContDB2 in terms of exposure to noise outside of military service. However, it seems likely that the exposure of the former during military service far outweighed their exposure during other work or leisure activities (Jokel et al., 2019).

## Summary and Conclusions

A new approach to the diagnosis of M-NIHL, based on the use of MLPs, was developed and evaluated. The approach was applied to databases containing the ages and audiograms of individuals claiming compensation for NIHL sustained during military service, who were assumed mostly to have M-NIHL, and control databases with no known exposure to intense sounds. The MLPs were trained so as to classify individuals as belonging to the exposed or control group based on their audiograms and ages, thereby automatically identifying the features of the audiogram that provide optimal classification. Two databases (noise-exposed and nonexposed) were used for training and validation of the MLPs and two independent databases were used for evaluation of the trained MLPs. The best-performing MLP, denoted MLP(18), was one trained to identify whether or not an individual had M-NIHL based on age and the audiogram for both ears. For the test databases, MilDB2 and ContDB2, this achieved a sensitivity of 0.986 and a specificity of 0.902, giving an overall accuracy markedly higher than for the M-NIHL (2020) and rM-NIHL methods. The MLP(18) method also gave high positive predictive values, even when the prevalence of M-NIHL among the population of claimants for compensation for M-NIHL was assumed to be unrealistically low.

It can be concluded that the M-NIHL (2020), rM-NIHL, and MLP(18) diagnostic methods all give PPV values above 0.5 when plausible prevalence values are assumed, thereby satisfying the balance of probabilities required in a medicolegal context, but that the MLP(18) method is more robust and has greater specificity than the M-NIHL (2020) and rM-NIHL methods.
